# High-Frequency Ultrasonic Spectroscopy of Structure Gradients in Injection-Molded PEEK Using a Focusing Transducer

**DOI:** 10.3390/s23146370

**Published:** 2023-07-13

**Authors:** Jannik Summa, Moritz Kurkowski, Christian Jungmann, Ute Rabe, Yvonne Spoerer, Markus Stommel, Hans-Georg Herrmann

**Affiliations:** 1 Fraunhofer Institute for Non-Destructive Testing IZFP, 66123 Saarbrücken, Germany; christian.jungmann@izfp.fraunhofer.de (C.J.); ute.rabe@izfp.fraunhofer.de (U.R.); hans-georg.herrmann@izfp.fraunhofer.de (H.-G.H.); 2 Leibniz-Institut für Polymerforschung Dresden e.V., 01069 Dresden, Germany; kurkowski@ipfdd.de (M.K.); spoerer@ipfdd.de (Y.S.); stommel@ipfdd.de (M.S.); 3 Institute of Materials Science, TU Dresden, 01062 Dresden, Germany; 4 Chair for Lightweight Systems, Saarland University, 66123 Saarbrücken, Germany

**Keywords:** high-frequency ultrasonic testing, property imaging, PEEK, morphological gradient

## Abstract

For high-performance thermoplastic materials, material behavior results from the degree of crystallization and the distribution of crystalline phases. Due to the less stiff amorphous and the stiffer and anisotropic crystalline phases, the microstructural properties are inhomogeneous. Thus, imaging of the microstructure is an important tool to characterize the process-induced morphology and the resulting properties. Using focusing ultrasonic transducers with high frequency (25 MHz nominal center frequency) enables the imaging of specimens with high lateral resolution, while wave propagation is related to the elastic modulus, density and damping of the medium. The present work shows experimental results of high-frequency ultrasonic spectroscopy (HF-US) applied to injection-molded polyether-ether-ketone (PEEK) tensile specimens with different process-related morphologies. This work presents different analysis procedures, e.g., backwall echo, time of flight and Fourier-transformed time signals, facilitating the mapping of gradual mechanical properties and assigning them to different crystalline content and morphological zones.

## 1. Introduction

Since the mechanical properties of semi-crystalline polymers are strongly affected by their microstructure and morphology, several methods have been developed for their characterization. Methods such as microscopy or X-ray diffraction, which can be used to resolve crystal structures inside the sample [1], are currently used for characterization. However, these require time-consuming and, in particular destructive preparation steps. Hence, a non-destructive testing method for rapid volume inspection, which facilitates the spatially resolved characterization of the semi-crystalline morphology, is of great interest. The present work takes up the well-known method of the HF-US pulse-echo technique and the analysis of frequency-dependent attenuation and investigates their suitability for characterization and mapping heterogeneous and gradual properties in the semi-crystalline polymer PEEK. This semi-crystalline polymer is known to form crystal phases with anisotropic spherulites, as illustrated in [1].

High-frequency ultrasonic testing (HF-US, 25 MHz nominal center frequency) is promising since acoustic waves interact with the propagating medium [2] consisting of amorphous material and spherulites. When the attenuation per wavelength is small, the acoustic measurements of sound velocity *c* and attenuation *a* can be related to the complex mechanical moduli *M′* (storage modul) and *M″* (loss modul) as follows [3]:

(1) $$M^{\prime }=\rho c^{2},\;M^{\prime \prime }=\frac{\rho c^{3}a}{2\pi f},\;c=\frac{\lambda }{f}$$

with *ρ* being the material density, *f* the wave frequency and *λ* the corresponding wavelength. Furthermore, for an incident wave propagating from one medium into another, the amplitude ratio of reflected to incident wave can be expressed by the acoustic impedance *Z*_1_ and *Z*_2_ of both media [4]:

(2) $$R=\frac{{\left(Z_{2}-Z_{1}\right)}^{2}}{{\left(Z_{2}+Z_{1}\right)}^{2}},\;Z_{i}=\rho_{i}c_{i}$$

with *ρ* and *c* again being the density and sound velocity of each medium.

Biwa [5] and Wu [6] report on the frequency-dependent interaction of acoustic waves with spherulites that can be measured by means of ultrasonic velocity and attenuation. Additional effects have to be considered since the attenuation of the ultrasonic wave is sensitive to the ratio of particle radius to wavelength as well as to particle concentration [5,7]. Following their conclusions, sound attenuation has a non-monotic trend if the ratio of particles to wavelength is large. The opposite is reported if the particle radius to wavelength ratio is small. In that case, the sound attenuation decreases with increased particle quantity since the effect of increased volume fraction of elastic glass particles predominates. These conclusions stem from studies on various polymers with glass particles of defined sizes from 22 µm to 45 µm in diameter and with user testing frequencies of 1 MHz to 6 MHz. These results agree with Wu et al. [6], who report the nonlinear sound attenuation as a function of the particle size and quantity in the frequency range from 1.5 MHz to 10 MHz. It is noteworthy that the reported interaction was observed, although the particles were smaller than the diffraction-limited theoretical resolution (ultrasonic wavelength approx. 250 µm) at 6 MHz or 10 MHz, respectively. Additionally, theoretical models [8,9,10] have been developed to numerically describe the sound attenuation considering grain size distribution and the wavelength. The proposed approach by Arguelles et al. [9] describes the sound attenuation $\alpha_{LL}$ as a function of the longitudinal wavenumber k_L_, the covariance between the emitted and the scattered wave M as well as the spatial correlation function *n* of the scattering angle $\theta_{ps}$ [9]:

(3) $$\alpha_{LL}=\frac{k_{L}^{4}\pi }{4\rho^{2}c_{L}^{4}}\int_{0}^{\pi }n(\theta_{ps})M(\theta_{ps})\sin\theta_{ps}d\theta_{ps}$$

Hereby, the spatial correlation function *n* depends on the grain size distribution. The applicability is questionable here since the model coefficients are unknown for a non-isotropic semi-crystalline material. One of their conclusions is that the longitudinal attenuation increases with increasing mean grain size. Others report that attenuation mainly comes from absorption associated with the viscoelasticity of the amorphous phase [11].

The previous works agree with [3], who observed the sound attenuation during the crystallization of low-density polyethylene. Based on the experimental results, the sound attenuation increased with increasing particle size and quantity. The authors of [3] add that crystallites or spherulites behave similarly to glass particles since the crystallites contribute to the scattering of acoustic waves. However, they did not discuss a possible effect that may come from a chain orientation. For the latter, an approach can be found to account for particle orientation in the work by Dunn and Ledbetter [12,13].

In addition, imaging ultrasound techniques, e.g., HF-US [14] or scanning acoustic microscopy (SAM), have to be addressed. Hereby, the recorded amplitude-time signals are displayed two-dimensionally over a scanned surface [15]. The mapping of the scanned surface can also be performed according to the frequency spectrum or the frequency of maximum amplitude [16]. By assigning the amplitude-time signals to the scanned surface (x, y) and the time (z), three-dimensional imaging of the microstructure is possible [17]. According to [15], attention should be paid to the decreasing lateral resolution with increasing depth. Applying a SAM with a focusing transducer at 50 MHz to differently cooled PEEK samples, Simonin et al. [15] were able to infer different mechanical properties. Based on the amplitude heights, slowly cooled PEEK samples suggested higher mechanical properties than fast-cooled ones. The presence of distributed high-amplitude zones indicated crystalline phases. It follows, for mapping techniques, that the amplitudes bear information on the mechanical properties [18], which can be a viable indicator for crystallinity.

## 2. Materials and Methods

The morphology in semi-crystalline thermoplastics such as PEEK has a significant influence on mechanical properties [1]. In the injection molding process, the morphology results from the process conditions and can be specifically influenced. Different flow and temperature conditions lead to strong local differences in the morphology of the injection-molded part [19,20]. Shear-induced and thermally induced crystallization take place competitively and form geometrically different structures. In contrast to thermally induced spherulites [21], shear-induced crystals exhibit anisotropic mechanical properties [22]. Due to the stiff polymer chains of PEEK, this shear-induced anisotropy is particularly pronounced due to the stiffer and more elastic behavior in the chain direction. Five distinct layers are observed in injection-molded parts [19]. In the two skin layers, which are located at the part’s surface, the hot melt touches the colder mold and cools rapidly. Due to the high cooling rate, orientations of the polymer chains freeze, and a predominantly amorphous state is formed. Between this first-solidified skin layer and the sample core, the melt is sheared by the cross-sectional constriction due to the higher flow velocity. Thus, two shear layers with chains oriented in the flow direction are formed. The morphology here is determined by shear-induced crystallization, which exhibits anisotropic mechanical and, thus, anisotropic ultrasonic properties. As soon as the mold is completely filled, the core layer in the middle of the part cools slowly without shear stress. Predominantly thermally induced spherulites are formed, which exhibit almost isotropic properties and differ in spherulite size. Subsequent annealing above the glass transition temperature and below the melting temperature can reduce residual stresses and increase the degree of crystallization in the outer zones [23].

**Figure 1:**
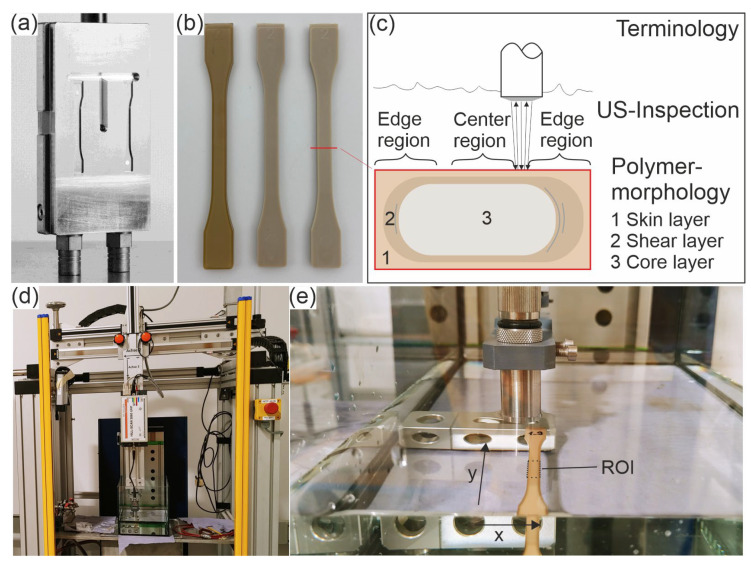
(**a**) Injection mold with two sample cavities, (**b**) PEEK samples 'cold-u’ (left), ‘hot-u’ (middle) and ‘hot-a’ (right), (**c**) illustration of the terminology of inspected regions with US and the morphological layers, (**d**) experimental setup with water bath and Hillger HILL-SCAN 3060 as pulser/receiver on a three-axes manipulator, (**e**) focusing transducer scanning the ROI of a PEEK sample.

For the investigations, tensile test specimens made of PEEK (VESTAKEEP 2000 G, Evonik, Essen, Germany) of type 1BA (specimen thickness: 2 mm) are produced according to DIN EN ISO 527 [24] in an injection molding process (Allrounder 420C, Arburg, Loßburg, Germany). The melt temperature at which the PEEK is injected into the mold is 390 °C. A mold with two cavities for the specimens and a sprue in the middle is used (see Figure 1a). In this work, only samples from the left cavity are examined. The mold temperature is varied to realize significantly different cooling and shear rates and, thus, different morphologies in the specimen. Subsequently, one type of specimen (see Table 1) is annealed above the glass transition temperature of 147 °C. This enables subsequent crystallization and reduction of residual stresses. Exemplary samples of each type are displayed in Figure 1b.

High-frequency ultrasonic testing is performed in a water bath. Besides the water bath, the main components of the system are a three-axes manipulator with an EPOS controller, a HILL-SCAN 3060 UHF (by Hillger, Braunschweig, Germany) as the pulser/receiver and a SPEC m3i 4140 card (by Spectrum Instrumentation GmbH, Grosshansdorf, Germany). The experimental setup is shown in Figure 1d,e.

The focusing ultrasonic transducer has a center frequency of 23.4 MHz (called 25 MHz in this work). The transducer (IAPF-F25.6.1, GE Sensing & Inspection Technologies GmbH, Pforzheim, Germany) is driven using the pulse-echo technique. The transducer is adjusted to the interface between water and the peek sample with a distance equal to its focal length of 24.6 mm. For measurements, the transducer scans the surface over the defined region of interest (ROI), shown in Figure 1e. The latter is 6 mm in the scan axis across the width of the sample and 10 mm in the index axis, which corresponds to the length direction of the samples. Table 2 summarizes all relevant testing parameters.

The thickness inspection of the samples is carried out using a confocal distance sensor system (sensor head CL-P030, control unit CL-3000, optical unit CL-P030N, calibration device OP88285, Keyence Deutschland GmbH, Neu-Isenburg, Germany). Hereby, two confocal sensor heads, each measuring the distance to the sample, face each other. Again, a two-axis manipulator facilitates the scanning movement of the probes over the ROI on the sample.

For the microscopy analysis, 10-µm-thick cross-sectional thin cuts were taken from the middle of the measuring length perpendicular to the flow direction of the melt using a Rotary Microtome RM2265 (Leica, Wetzlar, Germany). These are then investigated with an Axio M2m light microscope (Zeiss, Oberkochen, Germany) under polarized light.

## 3. Results

### 3.1. Material Characterization with Ultrasonic C-Scan

**Figure 2:**
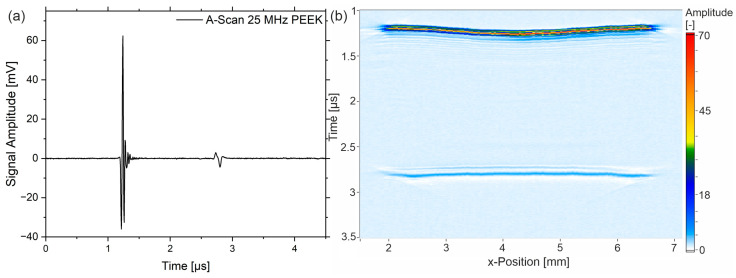
(**a**) A-scan with the sharp peaks of entrance echo and backwall echo and (**b**) B-scan over the width of a PEEK sample of type ‘cold-u’.

The ultrasonic investigations are carried out using a focusing ultrasonic transducer with a 25 MHz nominal center frequency. Due to the broadband excitation, the emitted wave is very sharp in the time domain. Figure 2a depicts the A-scan showing the sharp peak at the interface water–PEEK (entrance echo, *EE*) at approximately 1.2 µs and the backwall echo (*BE*) at approx. 2.9 µs for a PEEK-sample of the type “cold-u”. The shape of the *BE* differs from the *EE* due to the attenuation and scattering phenomena as the wave interacts with the propagation medium. Additionally, the B-scan over the width of the sample is given.

**Figure 3:**
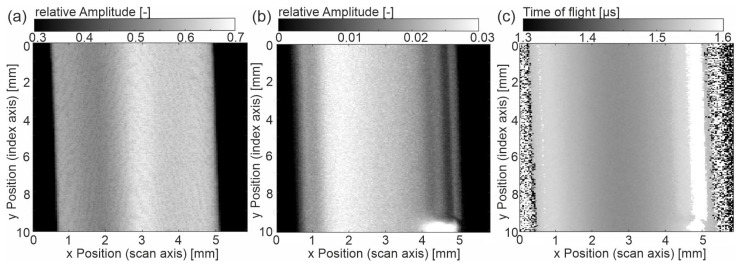
Sample ‘cold-u’ (**a**) C-scan of the amplitude at entrance echo, (**b**) C-scan of amplitude at backwall echo, (**c**) C-scan of the time of flight from entrance to backwall echo.

The basic data analysis in the following is based on the A-scan since the height of EE and BE, as well as the time between them (ToF: time of flight), is displayed. The B-scan gives the primary signal with respect to the width of the sample. Figure 2b shows that the sample ‘cold-u’ has a topographical difference between the center and the edge region, which can also be seen from the thickness measurements (Figure 6a). This change in thickness is due to plastic shrinkage during cooling in the injection molding process. Figure 3a shows the C-scan of the sample, where Figure 3b,c depicts the amplitude of the BE and the ToF. The latter are not classic C-scans since no gates are set. However, the images depict the information of the scanned surface and will be referred to as C-scans here.

The entrance echo (Figure 3a) shows a gradient in the reflected amplitude. Especially on the left side of the center, the amplitude is reduced. In addition, a fine line pattern is noticeable, showing lines of reduced amplitude height. The reflected amplitude is a superposition of the topography and local heterogeneous properties. The topography contrast results from the angle between the incident wave and the surface. Note that the inclination angle does not exceed 2.4°. The height difference does not affect the signal since the whole surface lies within the focus tube of the focused US beam. Secondly, different material properties at the surface cause different acoustic impedances, thus resulting in different values for the reflected US wave (see Equation (2)).

In the backwall echo (Figure 3b), the gradient in the x direction over the width of the sample is also visible. In contrast to the entrance echo, the maximum amplitude is shifted to the left, and the intensity distribution is slightly different. The edge appears grey. Following the x-direction towards the center, a darker line occurs, indicating the positions of lowest amplitude at approximately 0.3 mm distance to the edge on both sides. These lines correlate with the morphology (see interpretation of microscopy images, Figure 7). Note that the bright area at the right bottom comes from an air bubble on the backside of the sample. The time of flight (ToF) also shows the gradual difference between the center region and the edge region. The lowest ToF is found in the center, with the transit time increasing toward the edge regions. The latter is related to the sample thickness, which also increases from the center to the edges (Figure 6a).

**Figure 4:**
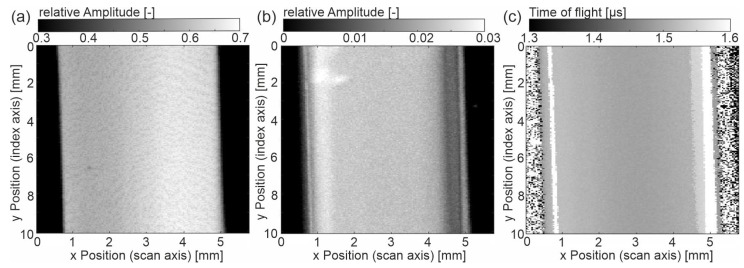
Sample ‘hot-u’ (**a**) C-scan of the amplitude at entrance echo, (**b**) C-scan of amplitude at backwall echo, (**c**) C-scan of the time of flight from entrance to backwall echo.

**Figure 5:**
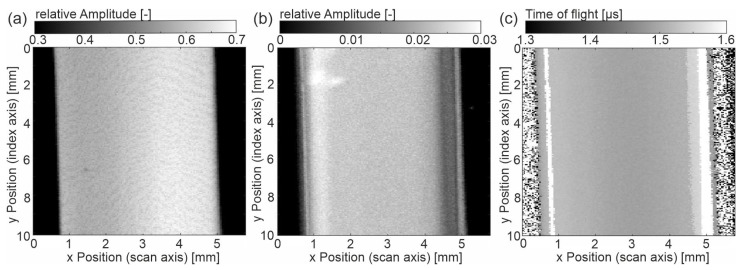
Sample ‘hot-a’ (**a**) C-scan of the amplitude at entrance echo, (**b**) C-scan of amplitude at backwall echo, (**c**) C-scan of the time of flight from entrance to backwall echo.

To better understand these observations, additional results on exemplary samples ‘hot-u’ and ‘hot-a’ are given in Figure 4 and Figure 5. Again, they show the entrance echo (EE), backwall echo (BE) and time of flight (ToF).

The entrance echo of ‘hot-u’ (Figure 4) is comparable with the one of ‘cold-u’, wherein the relative amplitude is increased, and the gradient is less pronounced. In contrast, the EE of the sample ‘hot-a’ is mostly homogeneous. The relative amplitude is highest here. In both EE images, line patterns are still visible. With respect to the BE and the ToF images, it seems that the gradients have strongly been reduced due to the hot tooling (‘hot-u’) and further due to annealing (‘hot-a’). The following features are still noticeable. In the backwall echoes, the center is mainly homogeneous. However, areas with lower amplitude appear close to the left and right edges. This agrees well with the ToF images, where bright stripes (approximately 0.3 mm to the sample edges on both sides) indicate the ultrasonic transit time is approximately 1.6 µs. In Figure 5a,b black dots appear (x/y: 1 mm/5 mm and 1.5 mm/3 mm), denoting low amplitude values. These artifacts come from air bubbles on the top surface of the sample.

**Figure 6:**
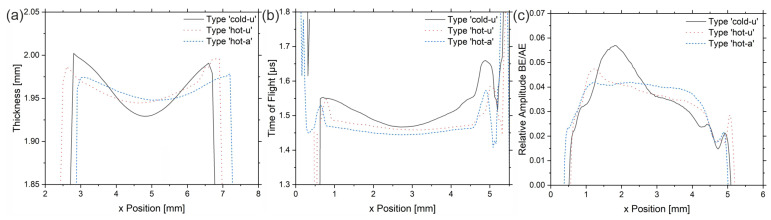
(**a**) Mean thickness over sample width, (**b**) Time of flight (ToF) from entrance to backwall echo, (**c**) relative amplitude of backwall echo and entrance echo.

Comparing the three samples with different manufacturing histories, the outcome is evident that the gradient of the sample ‘cold-u’ reduces towards high-temperature processed samples ‘hot-u’. The acoustic or mechanical properties become increasingly homogeneous if additionally annealed (‘hot-a’). It is worth mentioning that the sample ‘hot-a’ has a homogeneous entrance echo with no visible topography effect, although its topography is evident from Figure 6. This observation demonstrates that the topography vanishes for an inclination angle below 1.5° (‘hot-a’). Besides the entrance echo, the same trend applies to the time of flight as well as the wave attenuation from the entrance to the backwall echo. Considering Figure 6b and Table 3, it becomes evident that the transit time (ToF) and the calculated ultrasonic velocities change in the same manner. However, from Figure 6c, the attenuation seems higher for the sample ‘hot-u’.

Following the theory, the storage modulus of crystalline PEEK, thus also the sound velocity, is higher than for amorphous PEEK. The lower attenuation of the *BE* and the higher sound velocity (lower ToF) prove the increase in crystallinity from ‘cold-u’ to ‘hot-u’ to ‘hot-a’. Since the attenuation decreases with increasing crystallinity, the effects due to the increasing portion of the ‘rigid’ phase (see [5]) outweigh any other. The observed lines near the sample edges testify to high attenuation (low amplitude in *BE*) and low US velocity (high ToF). From the microscopy images, it can be seen that there is no dramatic loss in crystallinity in this region. It follows that the observed measuring effects have to be caused by an orientation effect due to the anisotropic properties of PEEK, which proves the assignment to the shear zone. For this purpose, [25] can be consulted, showing that the shear layer exhibits a high orientation while the core does not.

**Figure 7:**
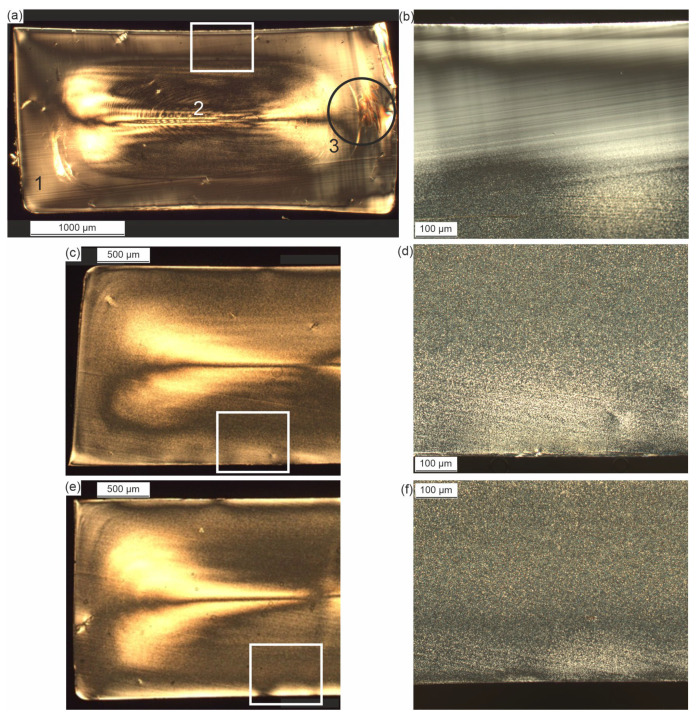
Polarized microscopy of thin films in the cross-sectional area of (**a**) cold-processed sample ‘cold-u’ with skin layer 1, core layer 2 and shear layer 3 with (**b**) detailed view of edge near zone; (**c**) hot-processed sample ‘hot-u’ with (**d**) detailed view of edge near zone; (**e**) hot-processed sample ‘hot-a’ with (**f**) detailed view of edge near zone.

With the information from the backwall echo, the waves have passed through the entire sample and can be seen as an integral result. Considering the literature, it is plausible that the differences in the US images originate from the different morphological states of the samples ‘cold-u’, ’hot-u’ and ‘hot-a’. Thus, images of polarized microscopy are additionally considered in Figure 7.

The injection-molded 1BA tensile bars were examined for their morphology properties. Figure 7a shows the micrograph of the cross-section of ‘cold-u’, which is perpendicular to the flow direction (see Figure 1b). It is seen that the specimen thickness changes across the width of the specimen. The reason for this dimensional change is the material shrinkage due to the process. This change in thickness can also be seen in the thickness measurement and Figure 2b). However, due to the absence of specimen preparation into thin sections, the effect there is less pronounced. From a morphological point of view, the specimen exhibits the typical layer characteristics of injection-molded specimens. As a result of the strong cooling, a pronounced skin layer (1) is visible, in which no structures could be resolved microscopically. The layer thickness increases towards the specimen corners. This could also be a reason for the signal gradient detected in the C-scan of the ultrasonic measurement (see Figure 3b). Adjacent to the skin layer, turbulences and flow lines (3) can be seen in some areas of the cross-section as a result of the shear conditions in the process. The flow lines extend in an eccentrically shaped way. However, a clearly defined shear layer, as described in the literature, is not evident from the image. In Figure 7a, amorphous regions are visible to the left (width: approx. 0.2 mm) and right (width: approx. 0.4 mm) of the flow line. In the C-scan (see Figure 3b), vertical lines are visible on this side, which have the same dimension as in the microscopy images. The line width on the left side (Figure 3b, x-position 1.3 mm) has the same width as the amorphous region in Figure 7a on the lower left near the flow lines. Therefore, it can be assumed that the lines in the ultrasound image correlate with the local morphology. In addition to the shear-induced flow lines, fine crystalline structures can be seen in the core layer (2), which cannot be defined more precisely due to their small size. The size of the crystalline structures increases from the skin to the core of the sample. Like the shear layer, the core is not symmetrical. This could be due to an asymmetric heat distribution in the injection mold due to the position of the cooling channels (see Figure 1a) or the heat input at the sprue. The asymmetry of the core layer is particularly pronounced in Figure 7a.

In addition to ‘cold-u’, the influence of the thermal process history (hot-u) and the subsequent heat treatment (hot-a) on the morphology was investigated. Figure 7b,d,f shows images of the skin layer of the investigated specimens. It is very clear that due to the lower cooling rate in the hot-u process, the skin is fine crystalline. The crystalline density in the skin appears to increase as a result of annealing and the resulting relaxation and rearrangement processes.

According to [5], a higher degree of crystallinity leads to a higher ultrasonic velocity and damping. Thus, the crystallinity of the sample ‘cold-u’ is the lowest, while the crystallinity of samples ‘hot-u’ and ‘hot-a’ is comparable (see Figure 7). Between those two, the ultrasonic velocity is higher for the sample ‘hot-a’, and the attenuation is higher for ’hot-u’. The microscopy images strongly support the explanation that the difference between these three sample types comes from the rapid cooling rate at ‘cold-u’, leading to a higher amorphous fraction—especially in the skin layer—and a stronger gradient. In the case of the hot processed samples, crystallization was not suppressed due to the lower cooling of the melt. Thus, they possess a higher crystallinity, while their morphology in the cross-sectional area (Figure 7) is dominated by a crystalline appearance. Additionally, the annealing process causes the lamellar-scale reorganization, growth and reorientation of spherulites, resulting in an increase in the crystal size and perfection [26]. Consequently, the crystallinity of the two samples, ‘hot-u’ and ‘hot-a’, behave differently in terms of the ultrasonic velocity and the attenuation. Following the literature [5,6,7], the higher attenuation in ‘hot-u’ agrees well with the smaller spherulites.

Approximately 0.3 mm close to the sample edges, one finds vertical lines with lower relative amplitude in the BE (Figure 6c), denoting higher damping as well as a higher ToF (Figure 6c). According to their position and the microscopy images, these areas can be assigned to the shear zone. Interestingly, the vertical lines in the images depicted in Figure 3b and Figure 4b occur wider on the right side than on the left, which is evident in microscopy only for the sample ‘cold-u’. Secondly, the EE of the US images suggests an asymmetric morphology distribution superimposed with topographical contrast. Both effects cannot be validated by microscopy for ‘hot-u’ and ‘hot-a’ since there is no evidence of flow lines or a gradient. Hence, further investigations are required to validate the effect using ultrasound.

### 3.2. Analysis of the Attenuation

**Figure 8:**
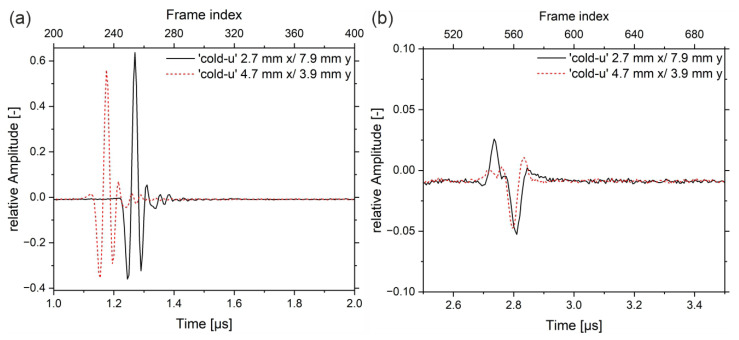
(**a**) entrance echo for sample ‘cold-u’ in the center (black curve) and the edge (red curve) and (**b**) corresponding backwall echoes.

Since the outer and inner regions of the samples show different US amplitude values, it is helpful to take a closer look at the waveforms or the shape of the peaks, respectively. Therefore, A-scans of the sample‚ ‘cold-u’ in the center zone (position x 2.7 mm/y 7.8 mm) and the edge zone (position x 4.7 mm/y 3.9 mm) are plotted in Figure 8.

At the edge, the signal passes predominantly through the non-crystalline skin layer, while at the center, the signal passes first through the skin layer, then through the crystalline core and shear layers, and finally through the second non-crystalline skin layer. In the entrance echo, the topography causes a slight delay of the peak between the edge area (red curve) and the center. Both signals have the same shape, whereas the max peak shows different amplitudes. The backwall echoes show different shapes compared to the EEs. During the interaction of the US wave with the propagation medium, it comes to a frequency-dependent attenuation, whereby the resulting superimposed wave of the backwall echo is different in intensity and shape from the EE. Further, the backwall echoes have different shapes. In the center (black curve), a double peak is evident at 2.73 µs and 2.77 µs, indicating higher frequencies. The frequency spectra are analyzed by means of the complex magnitude of the fast Fourier Transform (FFT) in the time ranges from 1 µs to 2 µs (frame index 200 to 400) and from 2.5 µs to 3.5 µs (frame index 500 to 700), respectively.

**Figure 9:**
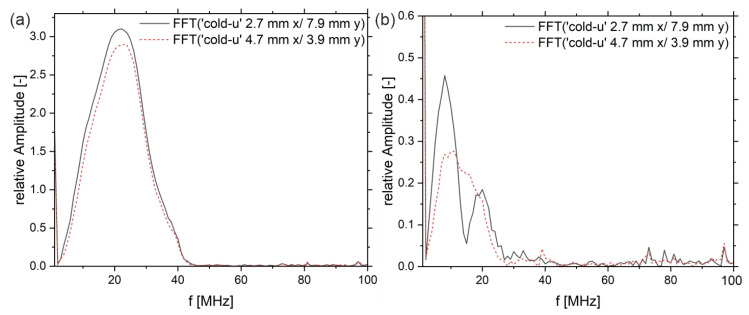
(**a**) complex magnitude of the FFT of the entrance echo for sample ‘cold-u’ in the core layer (black curve) and the skin layer (red curve) and (**b**) complex magnitude of the FFT of the corresponding backwall echoes.

In Figure 9a, the heights of the displayed spectra are different, wherein the intensity distribution in the frequency range from approx. 1 MHz to 42 MHz is similar. In the backwall echoes, first of all, the intensity of frequencies above 27 MHz vanished. Furthermore, differences can be observed. The black curve, which corresponds to the center region of the sample, shows two frequency bands with peak maxima at 8 MHz and 20 MHz and a local minimum at approx. 15 MHz. The red curve, on the other hand, shows a continuous spectrum from 1 MHz up to 28 MHz. Hence, in the edge region, the lower frequencies (3 MHz to 10 MHz) were attenuated significantly more than in the center of the sample (black curve). The same applies to frequencies above 20 MHz.

Again, according to the outlined literature (see introduction), frequency-dependent nonlinear damping of US waves indicates crystallinity in semi-crystalline polymers. Here, non-linear damping with maxima in attenuation at approximately 15 MHz and above 25 MHz can be seen (black curve). The red line, which is located close to the sample, shows the contrary. Thus, the differences in the frequency spectra of the backwall echoes appear to be a strong indicator of the material properties and the polymer structure, respectively. To examine this in more detail, the following is a comparison of the three sample types, ‘cold-u’, ‘hot-u’ and ‘hot-a’, by means of their damping spectra. These spectra show the frequency-dependent attenuation over the width of the samples. For this purpose, the quotient of the frequency spectra of the backwall echo and the entrance echo is considered and plotted logarithmically (see Equation (4)). It should be noted that the spectra are averaged in the longitudinal direction to achieve a better signal-to-noise ratio.

(4) $$D\left(f\right)=-\log\frac{I(f,\;Backwall)}{I(f,\;Entry)}$$

A conversion of the acoustic measurements, namely attenuation and sound velocity, into the mechanical moduli is not meaningful here since the density of amorphous and crystalline PEEK differs. However, the distribution of the spherulites in the volume is not known.

The damping spectra show a broadband damping in the range of 1 MHz up to 50 MHz, as well as the harmonics above. In addition to the broad background, the analysis is mainly based on the peaks that occur from 5 MHz up to 40 MHz, as well as their height and frequency.

**Figure 10:**
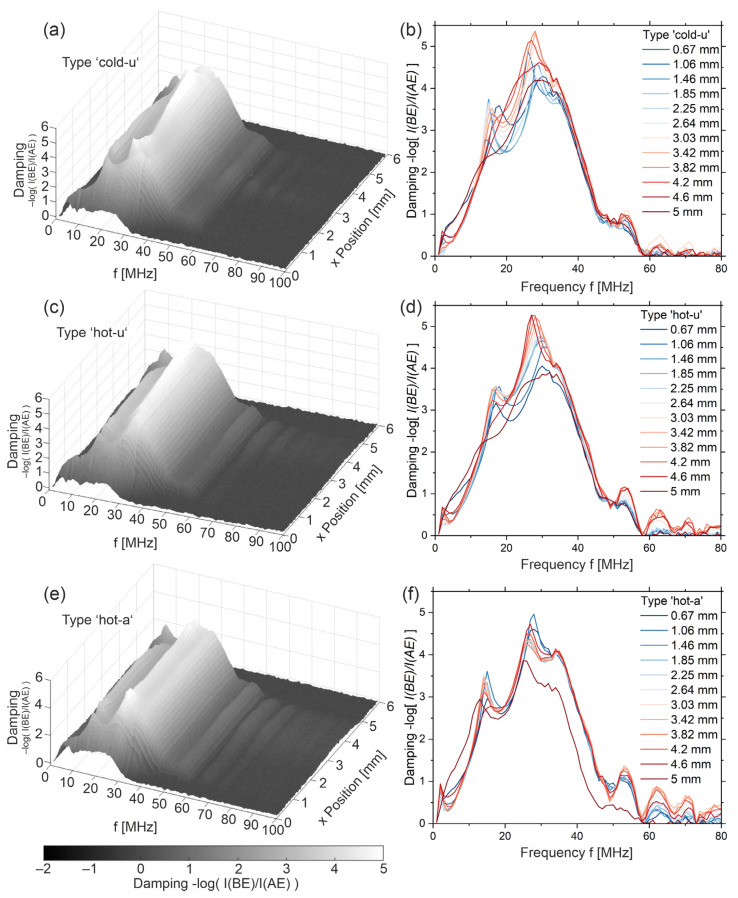
3D plot of damping spectra over the width of the samples (**a**) ‘cold-u’, (**c**) ‘hot-u’ and (**e**) ‘hot-a’ and the corresponding spectra at certain x-positions covering from left edge (0.67 mm) to right edge (5 mm) for (**b**) ‘cold-u’ (**d**) ‘hot-u’ and (**f**) ‘hot-a’.

The biggest differences can be seen with type ‘cold-u’. Here, the attenuation changes from the left to the right edge or from blue to red lines, respectively. Around the positions 1 mm and above 4 mm, the spectra show almost no peaks, while in the mid section, the position of the main peaks changes from 18 MHz and 25 MHz on the outside to 15 MHz and 28 MHz in the center area. The height also changes without an evident correlation to the peak position. These observations indicate a strong heterogeneity, which agrees well with the above-mentioned. Again, according to [5], the presence of peaks is assigned with higher crystallinity, while a broadband spectrum is assigned to higher amorphity. Increased scattering of the US wave occurs if the dimension and orientation of the crystalline phases (spherulite size, spherulite spacing) meet the direction and the wavelength or frequency (Equation (1)) of the US wave. In the case of a purely amorphous microstructure, uniformly distributed orientations and distances between neighboring polymer chains are present so that a statistically distributed attenuation results. Therefore, the results indicate a higher crystallinity in the center region. Additionally, in this area, peaks > 50 MHz are observable. Ultimately, Figure 10a indicates that the left side of the sample differs from the right. The area with lower damping coincides with the area of the backwall echo (Figure 3b), showing a higher amplitude. This could be explained by the non-uniform heat distribution in the injection mold.

For the second sample, type ‘hot-u’, the mentioned effects are likely but less pronounced. The underground, which speaks for the amorphous phase, is lower. The positions and heights of the peaks in the spectra vary less over the width of the sample. Thus, a more homogeneous and more crystalline microstructure can be concluded. This is confirmed by the microscopy images (Figure 7). Nevertheless, the maximum damping in the specimen x-position range from 0.6 mm to approx. 2.3 mm (left side) is at approx. 29 MHz to 30 MHz. On the right side (sample x-position 3.5 mm to 5 mm), the maximum damping lies at 26 MHz to 27 MHz and has a different shape. This could be related to the shear zone. In addition to crystallinity, the high damping may also be due to orientation effects. Seo et al. [22] have found that shear strongly promotes crystallization but also leads to the crystallites orientating according to the shear direction.

The annealed sample, type ‘hot-a’, shows mostly uniform attenuation spectra. Except for the edge, the magnitude of the attenuation is nearly congruent throughout the width of the sample with peaks at 14 MHz and 25 MHz as well as a shoulder at approx. 35 MHz. In addition, the peaks in the upper harmonics > 50 MHz are strongest here. Close to the edges, around 1.2 mm and 4.5 mm sample x-position (Figure 10e), the maximum attenuation values are higher. According to the location on the sample, these areas with the highest attenuation at 28 MHz can be assigned to the shear zone.

The comparison of the three sample types supports that the presence of peaks is indicative of higher crystallinity, while a broad spectrum is indicative of an amorphous polymer structure. Thus, the analysis of the attenuation spectra reflects very well that the crystallinity increases from ‘cold-u’ to ‘hot-u’ and ‘hot-a’. In addition, this method allows a statement about the spatial distribution of the amount of crystallinity over the sample. Interestingly, it follows from the spectra that higher crystallinity gives rise to harmonics above 50 MHz. Comparing the samples ‘hot-u’ and ‘hot-a’, the main peaks indicating higher crystallinity shifts from 18 MHz to 15 MHz and from 25 MHz to 28 MHz, while their amplitude heights change. A possible explanation is that the interaction of the ultrasonic waves with the spherulites depends on size and orientation since, for PEEK, the mechanical properties in the chain direction differ from those perpendicular to it.

**Figure 11:**
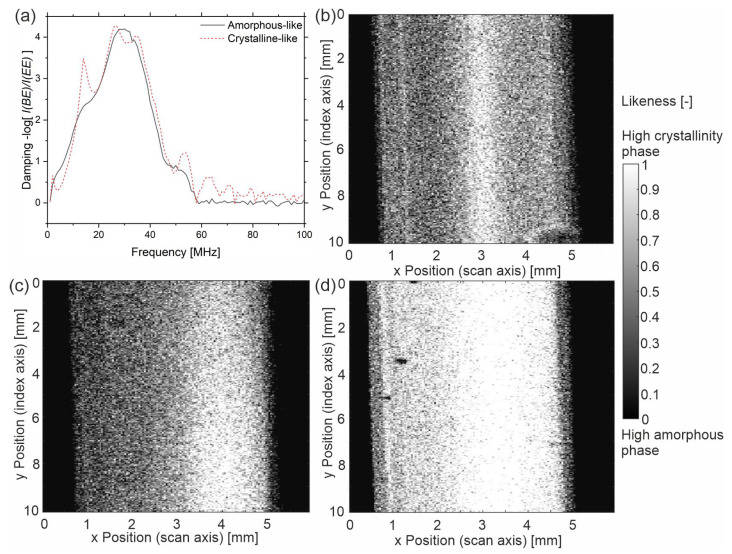
(**a**) amorphous-like and crystalline-like damping spectra; likeness to crystalline-like spectrum of (**b**) sample ‘cold-u’, (**c**) sample ‘hot-u’ and (**d**) sample ‘hot-a’.

To ultimately convert the results above into a C-scan, the following steps are necessary. Since there are no representative spectra for a perfectly crystalline polymer and a purely amorphous one, two spectra with extreme properties from our measurement series are defined as amorphous-like and crystalline-like. The respective spectra are taken from the US data located at the 5 mm sample x-position from ‘cold-u’ (amorphous-like) and at the 2.6 mm sample x-position from ‘hot-a’ (crystalline-like). Consequently, Figure 11 refers to the similarity or likeness instead of the absolute crystallinity. The likeness is determined numerically in terms of the volume fraction ϕ according to Equation (5) by means of the sum of least squares, where the summation is performed over the frequency.

(5) $$\underset{}{\varphi_{x,y}\to \min}\sum_{f}{\left({D\left(f\right)}_{a}\left(1-\varphi \right)+{D(f)}_{c}\cdot \varphi -{D(f)}_{x,y}\right)}^{2}$$

with *D*(*f*)*_a_* being the damping spectrum of the amorphous-like phase, *D*(*f*)*_c_* the damping spectrum of the crystalline-like phase, and *D*(*f*)*_x,y_* the damping spectrum at the measuring point (x,y).

The resulting images give the likeness to a spectrum, which is defined as ‘crystalline-like’. The sample of type ‘cold-u’ (Figure 11b) shows a low likeness, which indicates low crystallinity. In the center (core layer) and the bright lines at a distance of approximately 0.3 mm to both sample edges, the images indicate higher crystallinity. The latter was previously assigned to the shear layer. Figure 11d depicts the likeness of sample ‘hot-a’, indicating a high degree of crystallinity with higher degrees on the right side. Adjacent to the left skin layer, presumably the shear layer again, a bright line is found that indicates higher crystallinity. Note that the dark spots are artifacts due to air bubbles. Yet, these observations support the previous argumentation that crystallinity and homogeneity increase with increased processing temperature and annealing. However, the representation of the sample ‘hot-u’ contradicts this. Figure 11c follows a degree of crystallinity similar to the sample ‘cold-u’, which does not agree with the above results. Moreover, all three images denote dark areas at the sample edges, indicating lower crystallinity. This representation contradicts the observations made in Figure 7.

The authors conclude that geometry effects, such as presumably the edge effect causing the dark areas near the sample edges, must be taken into account. Likewise, not only the spectra for crystalline-like and amorphous-like phases have to be considered, but also effects due to orientation and size distribution. Ultimately, it can be concluded from the results that the method by means of the analysis of the damping spectrum of the backwall echo is suitable for imaging the degree of crystallinity and the homogeneity of semi-crystalline samples. However, further experiments are required to delineate other effects, such as orientation and size of crystallites.

### 3.3. Analysis of Local Amplitude Differences

The analysis of the damping spectra does not explain the observed line patterns that were visible in the C-scans of the entrance echo in Figure 3, Figure 4 and Figure 5a. A possible explanation is that this effect is caused by local differences, which vanish in the integral information given by the backwall echo. Accordingly, this section focuses on the local amplitude differences near the surface. As is visible in the A-scan (Figure 2), after the main peak, the amplitude clearly drops. Hence, the amplitude values with their coordinates on the *x-y* plane and depth *z* are normalized to the mean value of the *x-y* plane at their depth *z*.

(6) $$I_{normalized}^{x,y,z}=\frac{I^{x,y,z}}{\sum_{x,y}I^{x,y,z}}\cdot N^{x,y}$$

With *I^x,y,z^*, the amplitude at the measuring point (*x,y*) at the estimated depth *z* and *N^x,y^* the number of measuring points is a constant.

**Figure 12:**
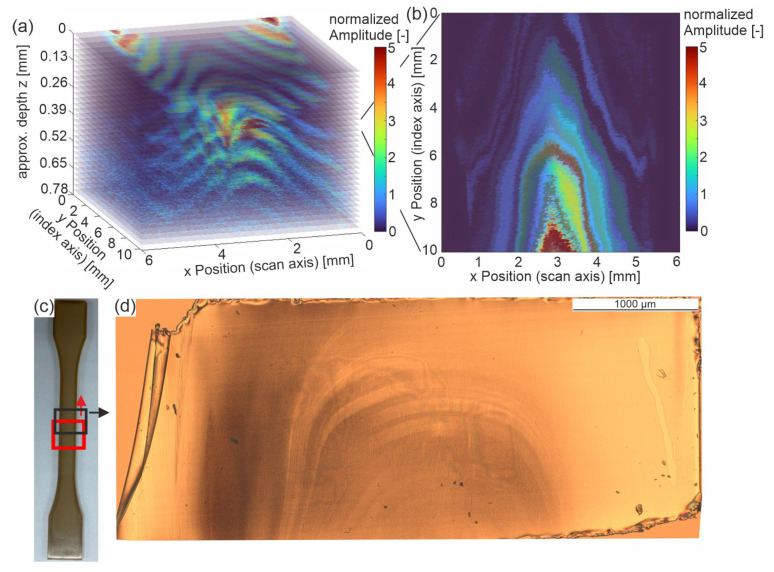
(**a**) 3D representation of normalized ultrasonic amplitude over depth of the sample ‘cold-u’, (**b**) corresponding 2D representation at approximated depth of 0.35 mm to 0.3 mm (**c**) sample positions of the HF-US measurement (red) and the polarized microscopy image (black), (**d**) polarized microscopy of a thin film at approximately 0.3 mm depth of sample ‘cold-u’.

Figure 12a shows a 3D plot of the normalized amplitude of the sample ‘cold-u’ referring to the sample position on the *x-y* plane and depth *z*. Note that the depth was estimated using the measured time of flight and with the assumed ultrasonic velocity of 2600 m/s. The depth and the time of flight are proportionally linked by the ultrasonic velocity. The magnitude or absolute value of the amplitude refers to the given color scale. The depth value was set to ‘0′ at the first occurrence of the entrance echo. It is evident from Figure 6a that the sample surface is higher at the edges than in the center. The same is seen here, as high amplitudes (red color) appear at the sample edges (y = 0 mm, x ≈ 0.5 mm and x ≈ 5 mm). From the course of the high amplitude values (red color), it is also evident that the sample surface in the center is deeper than at the edges. However, the figure shows the recorded data only up to a calculated depth of 0.78 mm.

For comparison, an image from polarized microscopy (Figure 12d) is depicted for approximately the region of the ultrasonic measurement at a depth of about 0.3 mm.

The measurement effect depicts areas with increased ultrasonic amplitude in certain areas of the sample. These areas differ in position with respect to the surface plane (x, y) as well as depth z resulting in line patterns. These patterns can also be seen in a C-scan of the normalized amplitude and in images from polarized microscopy of thin films (Figure 12d). The fact that the structures can be seen in both methods suggests that material differences are present and measurable. Since this effect has not been reported to the best of the author’s knowledge in the state of the art for semi-crystalline polymers, a clear conclusion on its cause is not feasible at present. However, the author’s first hypothesis is based on the literature regarding recrystallization being strongly affected by shear in the liquid state. Following this hypothesis, the displayed patterns arise due to melt flow in the injection molding process and thus indicate flow lines.

To clearly assign this effect and to assign it with material properties will be a task for future investigations. This requires further experimental studies on the behavior of high-frequency ultrasonic waves in semi-crystalline polymers.

## 4. Conclusions

Using high-frequency ultrasound with a focusing transducer with 25 MHz nominal center frequency applied to injection-molded PEEK samples resulted in 2D images (‘C-scans’) of the entrance and the backwall echo, as well as the detailed analysis of the wave information given by the backwall echo and the top-surface near region.

From the C-scans, it follows that different crystalline material states in the center and edge areas can be measured by means of acoustic properties and can be assigned to the core, shear and skin layers. The amplitude, as well as the calculated ultrasonic velocities, contributed to the conclusion that a higher fraction of amorphous material is in the skin—contrary to the core layer. Furthermore, the height of the amplitudes and the US velocities strongly correlate with the processing history, such that the amplitude height and US velocity increased with increasing crystallinity from the cold-processed sample to hot processed to the hot-processed and annealed sample. These results fit well with the observations made by polarized light microscopy.

In the detailed analysis of the attenuation spectra, characteristic courses for amorphous and crystalline material could be observed by means of broad attenuation and sharp peaks, respectively. In addition, two different locations of the peaks in the frequency domain were found for samples with high crystallinity. The cold-processed sample showed high differences across the sample width. The spectra indicated a higher amorphous fraction near the skin and a higher crystallinity in the core of the sample. In contrast, the hot-processed samples showed higher homogeneity and sharp peaks in the ultrasonic spectra. Additionally, upper harmonics appeared in the spectra with higher crystallinity. Ultimately, the analysis of the damping spectra is a suitable tool to map the crystallinity and structural properties. The spectra provide detailed information on the mechanical interaction of the microstructure with the US waves. The mapping of the likeness to a spectrum in a crystalline area showed that the method is principally suitable for mapping crystallinity. However, further investigations on referential crystalline spectra and influences from orientation and crystallite size are required.

## Figures and Tables

**Table 1:** Sample designation and processing conditions.

Sample Designation	Mold Temperature	Annealing
cold-u	38 °C	-
hot-u	184 °C	-
hot-a	184 °C	200 °C, 2 h

**Table 2:** Testing parameters for high-frequency ultrasound.

**Sample rate**	**Averaging**	**Scan range**	**Scan increment**	
200 MS/s	2	6 mm scan axis (x) 10.04 mm index axis (y)	0.0392 mm scan axis (x) 0.04 mm index axis (y)	
**Scan type**	**Active filter**	**Pulse sequence**	**Damping resistor**	**Gain**
Ridge drive	none	10 kHz	30 Ohm	8 dB

**Table 3:** Calculated ultrasonic velocities from ToF measurement and thickness inspection.

X Position from Sample Edge	Sample ‘cold-u’	Sample ‘hot-u’	Sample ‘hot-a’
0.5 mm	2625 m/s	2673 m/s	2699 m/s
1 mm	2657 m/s	2679 m/s	2706 m/s
1.5 mm	2661 m/s	2683 m/s	2709 m/s
3 mm	2565 m/s	2659 m/s	2680 m/s
3.5 mm	2514 m/s	2649 m/s	2669 m/s
4 mm	2368 m/s	2548 m/s	2605 m/s

## Data Availability

The data presented in this study are available on request from the corresponding author. The data are not publicly available as the raw data format is of proprietary file type being the property of Fraunhofer IZFP.
